# The regulatory roles of RNA-binding proteins in the tumour immune microenvironment of gastrointestinal malignancies

**DOI:** 10.1080/15476286.2024.2440683

**Published:** 2024-12-24

**Authors:** Dongqi Li, Xiangyu Chu, Weikang Liu, Yongsu Ma, Xiaodong Tian, Yinmo Yang

**Affiliations:** a Department of Hepatobiliary and Pancreatic Surgery, Peking University First Hospital, Beijing, China; b Department of General Surgery, Beijing Friendship Hospital, Capital Medical University, Beijing, China

**Keywords:** RNA binding protein, mRNAs, ncRNAs, tumour immune microenvironment, immunotherapy, targeted therapy

## Abstract

The crosstalk between the tumour immune microenvironment (TIME) and tumour cells promote immune evasion and resistance to immunotherapy in gastrointestinal (GI) tumours. Post-transcriptional regulation of genes is pivotal to GI tumours progression, and RNA-binding proteins (RBPs) serve as key regulators via their RNA-binding domains. RBPs may exhibit either anti-tumour or pro-tumour functions by influencing the TIME through the modulation of mRNAs and non-coding RNAs expression, as well as post-transcriptional modifications, primarily N6-methyladenosine (m^6^A). Aberrant regulation of RBPs, such as HuR and YBX1, typically enhances tumour immune escape and impacts prognosis of GI tumour patients. Further, while targeting RBPs offers a promising strategy for improving immunotherapy in GI cancers, the mechanisms by which RBPs regulate the TIME in these tumours remain poorly understood, and the therapeutic application is still in its early stages. This review summarizes current advances in exploring the roles of RBPs in regulating genes expression and their effect on the TIME of GI tumours, then providing theoretical insights for RBP-targeted cancer therapies.

## 1. Introduction

Gastrointestinal (GI) cancers represent a group of malignancies with high incidence and mortality, which impose a significant global health burden [1,2]. The tumour immune microenvironment (TIME), comprising various immune cell populations from both innate and adaptive immunity along with immune regulatory factors, plays a critical role in tumour progression [3,4]. Recent studies have highlighted the strong correlation between TIME and clinical outcomes in patients with cancers. Immunotherapy has emerged as a promising treatment for GI tumours, and quickly gains prominence in the field of oncology [5–7]. However, a major challenge is that many patients undergoing immunotherapy develop resistance and experience rapid relapse, largely due to the interactions between GI tumours and the TIME, which are mediated by immunosuppressive elements like programmed cell death ligand 1-positive (PD-L1^+^) exosomes [8,9].

**Figure 1.**
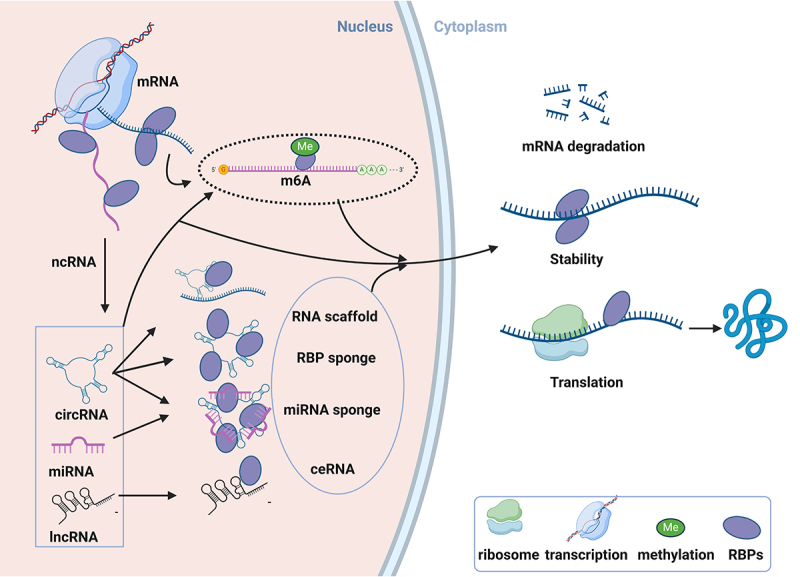
Post-transcriptional regulation of RNAs by RBPs. RBPs are involved in the regulation of mRNA stability, degradation, and translation through post-transcriptional modifications, including m^6^A methylation. Additionally, RBPs modulate the post-transcriptional processing of ncRNAs such as miRnas, lncRnas, and circRNAs by functioning as RNA scaffolds, RBP sponges, and miRNA sponges, influencing their activity and stability.

**Figure 2.**
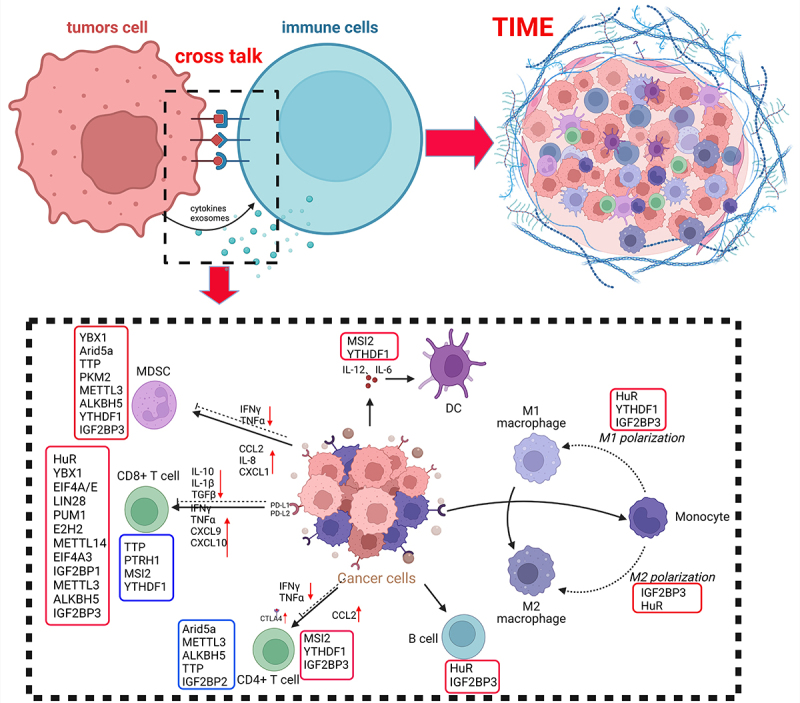
RBP-Mediated regulation of the TIME. RBPs play a key role in the post-transcriptional regulation of immune-related genes, facilitating communication between tumor cells and immune cells. This is achieved through the secretion of cytokines and exosomes, which alter the immune status within the tumor microenvironment, promoting immune evasion or enhancing immune responses depending on the context.

RNA-binding proteins (RBPs) are a highly conserved class of proteins essential for post-transcriptional regulation of genes [10]. They recognize mRNAs and noncoding RNAs (ncRNAs), or function as readers of N6-methyladenosine (m6A)-modified RNAs, to contribute to the malignant transformation of cancer cells (Figure 1) [11,12]. Notably, RBPs bind to RNAs through RNA-binding domains (RBDs) such as K homology (KH) domains, RNA recognition motifs, and zinc finger domains, forming intricate and overlapping post-transcriptional RNA regulons [13]. As a result, RBPs regulate oncogenes and tumour suppressor genes, influencing the TIME to promote GI tumours progression (Figure 2) [14]. In this review, we focus on the roles of RBPs in modulating the TIME in GI tumours, and explore the potential of targeting RBPs to enhance the efficacy of immunotherapy.

## 2. Dysregulation and dysfunction of RBPs in TIME

RBPs are primarily involved in the post-translational modification of RNAs, and any minor changes in RBPs affect downstream signalling pathways [15]. Therefore, dysregulation of RBPs is a driving force in the malignant transformation of tumour cells. RBPs are critical elements in transporting mRNAs from the nucleus to the cytoplasm, and both the 5*'*-UTR and 3*'*-UTR regions of mRNAs have binding sites for RBPs. Specifically, RBPs can interact with mRNAs at initiation, elongation and termination at the translational level to control genes expression [16]. For example, the Pumilio (PUM) protein family consists of several sequence-specific RBPs that inhibit translation and mRNAs degradation by binding to the 3’-UTR of mRNAs [17]. Further, RBPs also play intricate and dual roles in antitumor immunosurveillance within immune cells. For instance, Methyltransferase 3 (METTL3) deletion disrupted the m^6^A modification of Interleukin-1 Receptor-Associated Kinase 3 (IRAKM) mRNA, slowing its degradation, which in turn elevated IRAKM levels and impaired toll like receptor (TLR) signalling, thereby inhibiting macrophages activation [18]. Additionally, natural killer (NK) cells are known for their ability to recognize and eliminate tumour cells via natural killer group 2 member D (NKG2D) ligands, and are also influenced by RBPs such as insulin-like growth factor 2 mRNA-binding protein 3 (IMP3). For example, IMP3 suppressed the NKG2D ligand UL16-binding protein 2 (ULBP2) and indirectly downregulated MHC class I polypeptide-related sequence B (MICB), which facilitated tumour immune evasion [19]. Consequently, the TIME facilitates the selection of tumour cells that thrive within the limited resources of the microenvironment, accelerating tumour progression [20].

Accumulating evidence underscores the roles of RBPs dysregulation in tumorigenesis and progression through various mechanisms, including genetic mutations, epigenetic changes, and ncRNA-mediated regulation [21,22]. Even minor aberrations in RBPs can profoundly disturb cellular homoeostasis. For example, Human antigen R (HuR), a well-known RBP, has been implicated in promoting the uncontrolled proliferation of GI and other cancers. HuR not only drives chemoresistance but also facilitates the transmission of oncogenic signals within the tumour microenvironment (TME) by promoting the release of tumour-derived exosomes (TDEs) [23–25]. In gastric cancer (GC), exosomal microRNA-1246 (miR-1246) was found to be positively correlated with HuR expression [26]. Similarly, it was verified that colorectal cancer (CRC) cells secreted three times as many exosomes as normal cells, and this effect was also linked to HuR expression, and sliencing HuR significantly reduced exosome secretion in CRC cells [24]. Furthermore, hyperoxia-induced exosomal circHIF1A in cancer-associated fibroblasts (CAFs) enhanced immune escape in hepatocellular carcinoma (HCC) cells by upregulating PD-L1 expression in a HuR-dependent manner.

Recently, the concept of tunnelling nanotubes (TNTs) has also been proposed and progressively explored [27,28]. TNTs are intercellular conduits that supports communication between non-adjacent cells in the same tissue, importantly, TNTs allow transportation of a variety of cargoes, including small molecules (Ca^2+^ ions), macromolecules (nucleic acids, proteins, etc.) and even organelles (mitochondria, vesicles, etc.) [29,30]. For example, TNTs mediated the intercellular transfer of mutant RAS to induce intratumorally heterogeneity, resulting in more aggressive phenotypes in recipient cells [31]. Notably, TNTs was found mediating crosstalk between tumour cells and immune cells, helping them to overcome immunosurveillance to shape the TIME [32]. Lee et al. [33] found that the CMs of M0 and M1 macrophages induced immune stress responses to pancreatic cancer cells, which supported TNTs formation. This finding is valuable for the development of macrophage-based targeted cancer therapies. Therefore, a deeper understanding of the crosstalk between TIME and tumour cells could unlock new strategies for the clinical application of immunotherapy.

## 3. RBPs affect the TIME by regulating mRNA expression

RBPs regulate immune-related gene expression by recognizing and tightly binding to specific sequence elements of mRNAs, such as adenine and uridine-rich elements (AREs) and stem-loop structures. As a result, RBPs can enhance the stability of pro-oncogenic mRNAs, thereby promoting GI tumour progression by modulating the TIME (Table 1).

**Table 1.** RBPs influence the expression of mRNAs to regulate the GI TIME.

RBPs	mRNA types	Tumor	Effect	Researchers	Year
HuR	IAP1/2	PDAC	Carcinogenic	Lukosiute Urboniene et al.	2019 [34]
	PD-L1	PDAC	Carcinogenic Carcinogenic	Tao et al.	2019 [35]
	HSF1	CRC		Ren et al.	2022 [36]
YBX1	PD-L1	HCC	Carcinogenic	Tao et al.	2019 [35]
Arid5a	Ido1 Ccl2	PDAC	Carcinogenic	Hashimoto et al.	2022 [37]
eIF4A/E	ARF6 AMAP1	PDAC	Carcinogenic	Hashimoto et al.	2021 [38]
LIN28	Oct4	HCC	Carcinogenic	Patra et al.	2023 [39]
TTP	HO-1	CRC	Inhibitory	Seo et al.	2015 [40]
	PD-L1	GC	Inhibitory	Guo et al.	2018 [41]
	ICAM-1	CRC	Inhibitory	Seo GS	2015 [40]
PTRH1	PTRH1	PDAC	Inhibitory	Gao et al.	2023 [42]
MSI2	HMGB1	CRC	Inhibitory	Meng et al.	2024 [43]

*PDAC, pancreatic ductal adenocarcinoma; CRC, colorectal cancer; HCC, hepatocellular carcinoma; CA, colon adenocarcinoma; GC, gastric cancer.

Firstly, HuR is a widely expressed RBP in GI tumours and binds to transcripts of components of the TNF receptor (TNFR) complex, such as inhibitor of apoptosis protein 1 (IAP1) and inhibitor of apoptosis protein 2 (IAP2), which regulate NF-κB signalling downstream of multiple TNF superfamily receptors in immune cells [34,44]. It was found that HuR acted as a negative regulator of T-cell co-stimulation, potentially contributing to tumour immune evasion, but the exact role of IAPs in immune responses remains insufficiently studied [45]. In addition, Heat Shock Transcription Factor 1 (HSF1) was demonstrated a negative correlation with the expression of immunomodulators, including PD-L1, cytotoxic T-lymphocyte-associated protein 4 (CTLA4), and PD-L2. Notably, HuR could directly bind to the coding sequence of HSF1 mRNA, promoting the malignant phenotype of CRC [36].

Further, YBX1 (YB1), another pro-oncogenic RBP, is associated with poor prognosis of GI tumours. YB1 could not only bound to the PD-L1 promoter to increase its expression, but also suppressed the secretion of chemokines like interleukin 10 (IL-10), interleukin 1 beta (IL-1β), and transforming growth factor beta (TGFβ), which fostered an immunosuppressive microenvironment to facilitate immune evasion of HCC [35]. They also found that knocking down YB1 would reverse this immunosuppressive environment, leading to the inhibition of myeloid-derived suppressor cells (MDSCs) and regulatory T (Treg) cells, activation of CD8^+^ T cells, and increased secretion of cytokines such as interferon-gamma (IFNγ) and tumour necrosis factor-alpha (TNFα) [35]. Similarly, Arid5a stabilized CCL2 mRNA, enhancing the infiltration of immunosuppressive cells such as Tregs and MDSCs [37]. Notably, RBPs can regulate the production of immunosuppressive metabolites to help tumour cells immune escape. For example, Arid5a was also verified stabilizing indoleamine 2,3-dioxygenase-1 (IDO1) mRNA, thereby promoting Treg cells differentiation and activation by reducing intratumoral tryptophan levels, and facilitating IDO1’s inhibitory effects on effector CD4^+^/CD8^+^ T cells [37]. Further, LIN28 had been shown to protect tumour-initiating cells in HCC by directly promoting the translation of octamer-binding transcription factor 4 (OCT4), leading to increased levels of the immunosuppressive metabolite kynurenine, thereby aiding in immune escape [39].

Additionally, eukaryotic translation initiation factor 4a (EIF4A) and eukaryotic translation initiation factor 4e (EIF4E) regulated mRNAs stability and promoted the translation of small GTPase ADP-ribosylation factor 6 (ARF6) and its downstream effector multiple-domain Arf-GAP protein 1 (AMAP1) in pancreatic ductal adenocarcinoma (PDAC), driving PD-L1 recycling and surface expression following receptor tyrosine kinase (RTK) activation [38]. The PUM protein family, specifically PUM1, was also found binding and stabilizing nucleoplasmin3 (NPM3) mRNA, suppressing anti-tumour activity of T cells via PUM1/NPM3/PD-L1 axis in GC [46].

Interestingly, as described above, RBPs recognize effectors located in the 3’-UTR region of mRNAs. After binding to mRNAs and forming the RNP complexes, RBPs also use the cytoskeletal machinery as transport complexes to direct mRNAs to appropriate destinations, which affect tumour progression [47]. For example, IGF2BPs are highly conserved RBPs and are widely expressed in various cancers, including GI tumours. and are involved in regulating the localization of target RNAs, and their expression is usually associated with poor prognosis [48,49]. Musashi (MSI) proteins are a class of RBPs that play important roles in the translation process and are implicated in CRC and GC [43,50]. Similarly, knocking down MSI1 inhibits GC proliferation and colony formation [51]. In some young GC patients, the expression level of MSI1 is thought to be significantly correlated with the depth of tumour infiltration, lymph node metastasis and tumour stage [52].

Notably, RBPs not only function as oncogenic drivers of GI tumour progression but also as inhibitors of malignant phenotypes by binding to the 3*'*-UTR of mRNAs, facilitating the degradation of target transcripts (Table 1). For instance, Guo et al. [41] demonstrated that tristetraprolin (TTP) overexpression exerted anti-tumour effects in GC by enhancing the cytotoxicity of peripheral blood mononuclear lymphocytes (PBMLs). TTP increased T cells infiltration and strengthened anti-tumour immunity by reducing PD-L1 mRNA stability [41]. In CRC cells, TTP overexpression assisted haem oxygenase-1 (HO-1) in destabilizing intercellular adhesion molecule-1 (ICAM-1) mRNA at the post-transcriptional level, which was crucial for tumour-PBML interactions [40]. Peptidyl-tRNA hydrolase 1 homolog (PTRH1), another inhibitory RBP, suppressed PD-L1 expression by binding to the 3*'*-UTR of PD-L1 mRNA, thereby enhancing CD8^+^ T cell infiltration and improving their cytotoxic efficacy in pancreatic cancer [42]. Further, overexpression of PTRH1 combined with PD-L1 antibody treatment markedly reduced PDAC progression by lowering the proportion of Tregs. Similarly, Musashi-2 (MSI2) enhanced high mobility group box 1 (HMGB1) translation by binding directly to nucleotides 1403–1409 of HMGB1 3*'*-UTR, promoting dendritic cells (DCs) maturation, migration, and the infiltration of CD4^+^ and CD8^+^ T cells, which boosted immune infiltration in CRC [43].

In conclusion, RBPs play dual roles in modulating the TIME by either upregulating or downregulating target mRNA expression, significantly impacting GI tumour progression. Identifying key pro-oncogenic or anti-tumour RBPs is essential for advancing RBP-targeted therapeutic strategies.

## 4. RBPs affect the TIME of gastrointestinal malignancies by regulating the function of non-coding RNAs (ncRNAs)

NcRNAs, including microRNAs (miRNAs), long non-coding RNAs (lncRNAs), and circular RNAs (circRNAs), account for 98% of the human transcriptome and play critical roles in regulating the TIME, either by promoting or inhibiting tumour progression [53]. NcRNAs contribute to the phenotypic transition of tumour-associated macrophages (TAMs) from M2 to M1 polarization, modulate NK cells cytotoxicity, facilitate tumour immune escape, and influence the response to immunotherapy by regulating immune checkpoint proteins [54]. It is well established that RBPs function as either oncogenic or anti-tumour agents in GI tumours, in part by binding to ncRNAs and regulating their expression. This review highlights recent advances in understanding how RBPs modulate ncRNAs to influence the TIME and drive GI tumour progression (Table 2).

**Table 2.** RBPs interact with ncRNAs to regulate the GI TIME.

RBPs	Transcript	Tumor	Effect	Researchers	Year
LIN28A/B	let-7microRNA	HCC/CA	Tumor-promoting	Ma et al.	2021 [58]
HuR	miR-4312	PDAC	Inhibitory	Li et al.	2018 [62]
EZH2	LINC00152	CRC	Inhibitory	Ou et al.	2021 [69]
PKM2	HITT	GC	Inhibitory	Zhao et al.	2022 [71]
HuR	MIR155HG	HCC	Tumor-promoting	Peng et al.	2022 [67]
HuR	hsa_circ_0074854	HCC	Tumor-promoting	Wang et al.	2021 [81]
METTL3	circMYO1C	PDAC	Tumor-promoting	Guan et al.	2023 [84]
EIF4A3	circRHBDD1	HCC	Tumor-promoting	Cai et al.	2022 [82]
EIF4A3 hnRNPA2B1	circCCAR1	HCC	Tumor-promoting	Hu et al.	2023 [83]

*PDAC, pancreatic ductal adenocarcinoma; CRC, colorectal cancer; HCC, hepatocellular carcinoma; GC, gastric cancer; CA, colon adenocarcinoma.

### 4.1. MiRNA

MiRNAs are small non-coding RNAs (19–24 nucleotides) that suppress genes expression by binding to the 3’-UTR of target mRNAs [55,56]. Dysregulated miRNAs significantly contribute to the formation of the immunosuppressive TIME [57]. However, more specific mechanisms by which RBPs stabilize miRNAs and shape the TIME in GI tumours remain unclear.

There was evidence that Lin-28 homologs A (LIN28A) and B (LIN28B) were associated with poor prognosis in GI tumours [58]. LIN28A/B are let-7-specific RBPs that repress the expression of let-7 by binding to the conserved GGAG/GGUG motif or by post-transcriptionally inhibiting the maturation of let-7 precursors (pri and pre-let-7) through recruiting terminal uridylyl transferases TUT4 and TUT7 to polyuridylate pre-let-7 miRNA [59]. LIN28 overexpression enhanced the expression of oncogenes such as the high mobility group A2 (HMGA2), cell-myc (c-MYC), and Kirsten rat sarcoma virus (KRAS) by inhibiting let-7 biogenesis and function [60,61]. Further, the Lin28/let-7 axis is pivotal in facilitating tumour immune evasion. For example, LIN28 promoted PD-L1 expression through the LIN28/let-7/PD-L1 axis, enabling tumour cells to evade immune surveillance [39]. Moreover, LIN28 regulated the reprogramming transcription factors sex-determining region Y-box 2 (SOX2) and Oct4, which acted as enhancers of IDO1, producing the immunosuppressive metabolite kynurenine via targeting let-7 [39]. Interestingly, inhibiting Lin28B had been shown to improve the immunotherapeutic efficacy of glypican-3 (GPC3)-chimeric antigen receptor (CAR) T cells in HCC. Additionally, Li et al. [62] demonstrated that knocking down B-cell lymphoma 2 (Bcl-2)-associated athanogene 3 (BAG3) enhanced the loading of the miR-4312-containing miRNA-induced silencing complex (miRISC), inhibiting HuR’s cytoplasmic translocation and downregulating IL-8 expression.

Therefore, RBPs regulate miRNAs biological processes, including their activity and stability, by binding to specific sequences. The dysregulation of miRNA levels has widespread effects on gene expression and contributes to the formation of a suppressive TIME in GI cancers.

### 4.2. LncRNAs

LncRNAs, a subclass of ncRNAs longer than 200 nucleotides, play critical roles in regulating genes transcription, epigenetic modifications, and post-transcriptional processes. RBPs regulate the stability, translocation, and transcription of lncRNAs, thereby modulating tumour immunity, including antigen presentation, immune cells activation, and infiltration into GI tumour tissues [63,64]. Specifically, RBPs are involved in processes such as m^6^A modification, alternative splicing, and subcellular localization of lncRNAs [65]. Further, some core motifs, including U-rich sequences, AC repeat motifs, HUUWAAWA motifs, flanking base compositions, and RNA secondary structures such as hairpin stem-loop structures and dangling ends (unpaired nucleotides at the termini of double-stranded nucleic acids), are subject to recognize and regulate by RBPs [11,66]. Huang et al. [66] utilized enhanced crosslinking and immunoprecipitation (eCLIP) data to identify RBP-binding peaks on exonic and intronic regions of lncRNAs, which suggested RBPs critically shape lncRNAs fate.

Importantly, RBPs also participate in shaping the TIME of GI malignancies by modulating lncRNAs expression. For instance, Peng et al. [67] verified that lipopolysaccharide (LPS) upregulated METTL14, an RNA methyltransferase, promoting m^6^A methylation of the lncRNA MIR155 host gene (MIR155HG). Next, HuR stabilized MIR155HG, facilitating PD-L1 expression through the miR-223/signal transducer and activator of transcription 1 (STAT1) axis to facilitate immune evasion of HCC. Similarly, Zeist homologous enhancer 2 (EZH2) also binds to lncRNAs [68]. Ou et al. [69] demonstrated that EZH2 interacted with LINC00152 in GC to enhance C-X-C motif chemokine ligand 9 (CXCL9) and CXCL10 expression, ultimately suppressing CD8^+^ T cell infiltration. Importantly, they explored that LINC00152 interacted with EZH2 to inhibit CD8+ T-cell trafficking by the CXCL9, −10/CXCR3 axis, which promoted GC progression. Interestingly, the non-canonical RBP pyruvate kinase M2 (PKM2) is linked to metabolic regulation [70]. Zhao et al. [71] found PKM2 bound to the lncRNA HIF-1α inhibitor (HITT) at the transcriptional level, which reduced lactate production in GC cells, and promoted M1 macrophages polarization via a lactate-dependent non-cell autonomous mechanism.

Therefore, identifying the core motifs in lncRNAs recognized by RBPs is important to unravel their regulatory mechanisms, and pinpointing the binding sites between RBPs and lncRNAs is essential for understanding the expression of immune-related genes.

### 4.3. CircRNAs

CircRNAs are a prominent class of endogenously expressed ncRNAs that form covalently closed loops through reverse splicing. They play significant roles in cellular physiology, functioning as miRNA sponges, transcriptional regulators, RBP-binding molecules, templates for protein translation, and immune regulators [72]. Notably, RBPs are also critical in mediating the function of circRNAs, particularly by acting as scaffolds to regulate the expression of downstream targets [73]. Several predictive models, such as CRMSS, SSCRB, ASCRB, CircSSNN, and CRBP-HFEF, have been developed to identify RBP binding sites on circRNAs [74–78]. CircRNAs primarily function as competitive endogenous RNAs (ceRNAs), modulating miRNA activity by binding to miRNA response elements (MREs) [79].

CircRNAs are implicated in regulating M2 macrophage polarization, and this process is closely tied to RBPs. For instance, circASPH interacted with IGF2BP2 to enhance the stability of m^6^A-modified stimulator of interferon genes (STING) mRNA, enhancing exosomal STING to promote M2 macrophage polarization and accelerate CRC progression [80]. Wang et al. [81] also found that downregulation of hsa_circ_0074854 through its interaction with HuR inhibited M2 macrophage polarization, which was also achieved by affecting hsa_circ_0074854 transferred into HCC cells. Similarly, circRNAs also influence PD-L1 expression via RBPs. Cai et al. [82] demonstrated that circRHBDD1 acted as a scaffold to enhance the interaction between YTH N6-methyladenosine RNA binding protein 1 (YTHDF1) and phosphoinositide 3 kinase regulatory subunit 1 (PIK3R1) mRNA, regulating PIK3R1 mRNA translation in an m^6^A-dependent manner. Then, they found that eIF4A3 further upregulated circRHBDD1, hindering anti-PD-1 therapy via the circRHBDD1/YTHDF1/PIK3R1 axis in HCC. Moreover, circCCAR1 has been shown to promote resistance to anti-PD-1 immunotherapy in HCC through the circCCAR1/miR-127-5p/Wilms tumour 1-associating protein (WTAP) axis. Specifically, exosomal circCCAR1 was taken up by CD8^+^ T cells, leading to their dysfunction by stabilizing the PD-1 protein [83]. Interestingly, cyclized circRNAs also play key roles in suppressing tumour immunity. METTL3 was found inducing the circularization of circMYO1C, which enhanced PD-L1 mRNA stability by reading the m^6^A-modified site on PD-L1 transcripts, then promoted PDAC immune evasion [84].

However, it is still required to fully elucidate the mechanisms of RBP-circRNA interactions and their effects on the TIME in GI cancers, and potentially explore novel therapeutic strategies for GI malignancies.

## 5. RBPs influence the TIME of gastrointestinal malignancies through m^6^A modification of RNAs

The m^6^A modification is the most common RNA chemical modification in eukaryotic cells, regulating key processes such as RNA translation, localization, stability, and splicing [85]. m^6^A ‘readers’ recognize methylation on RNA and influence various physiological processes, including immune responses and tumorigenesis [86]. RBPs often act as m^6^A readers, binding to methylation sites on RNAs to modulate their metabolism [87] (Table 3).

**Table 3.** RBPs read m^6^A-modified RNAs to regulate the GI TIME.

RBPs	Transcript	Tumor types	Effect	Researchers	Year
YTHDF1	MHCII IL-12	GC	Inhibitory	Bai et al.	2022 [91]
	PD-L1	CA	Inhibitory	Han et al.	2019 [90]
YTHDF2	STAT1 IRF1	CRC	Carcinogenic	Wang et al.	2020 [92]
ALKBH5	PD-L1	ICC	Inhibitory	Qiu et al.	2021 [95]
		HCC	Carcinogenic	You et al.	2022 [94]
IGF2BP1	PD-L1	CA	Carcinogenic	Yao et al.	2024 [97]
IGF2BP2	circQSOX1	CRC	Carcinogenic	Liu et al.	2022 [98]
IGF2BP3	circCCAR1	HCC	Carcinogenic	Hu et al.	2023 [83]
IGF2BP2/3	IFIT1	CRC	Carcinogenic	Gao et al.	2023 [100]
IGF2BP2	circMYO1C	PDAC	Carcinogenic	Guan et al.	2023 [84]

*PDAC, pancreatic ductal adenocarcinoma; CRC, colorectal cancer; HCC, hepatocellular carcinoma; ICC, intrahepatic cholangiocarcinoma; CA, colon adenocarcinoma.

Among the most prominent m^6^A readers are the YTHDF family proteins, which contain a YTH domain and mediate specific cellular functions. YTHDF1 is central to RNA methylation, playing a pivotal role in both tumorigenesis and tumour immunity [88,89]. Han et al. [90] proved that YTHDF1 recognized and promoted the transcription of lysosomal cathepsins of DCs in colon cancer (CC). Notably, in YTHDF1-deficient (YTHDF1−/−) mouse models, they observed enhancing cross-presentation and priming of CD8+ cytotoxic T cells. Further, YTHDF1 deficiency in GC also led to the recruitment of mature DCs, increasing CD4^+^ and CD8^+^ T cell infiltration and secretion of interferon-gamma (IFN-γ) [91]. It’s reported that METTL3 and METTL14 are linked to poor immunotherapy responses in patients with CRC. However, YTHDF2 synergistically enhanced the therapeutic effect of targeting METTL3 and METTL14 by stabilizing signal transducer and activator of STAT1 and interferon regulatory factor 1 (IRF1) mRNAs, increasing the infiltration of cytotoxic CD8^+^ T cells and secretion of IFN-γ, CXCL9, and CXCL10 within the TME [92].

Further, alkylation repair homolog 5 (ALKBH5), an m^6^A demethylase, has been found to be downregulated in various cancers, contributing to tumour progression and modulating the TIME via m^6^A modification of mRNAs [93]. You et al. [94] demonstrated that ALKBH5 in HCC upregulated IL-8 expression and recruited PD-L1^+^ macrophages through the ALKBH5/MAP3K8/IL-8 axis, thereby promoting the formation of TIME. Additionally, ALKBH5 regulated TIME by inhibiting the infiltration of MDSC-like cells and promoting PD-L1 expression on monocyte/macrophage cells via YTHDF2 in intrahepatic cholangiocarcinoma (ICC) [95].

The IGF2BP proteins also act as m^6^A readers, recognizing m^6^A sites through their KH domain to enhance mRNA stability and translation, thus shaping the TIME [96]. Recent research uncovered a hydrophobic groove within the KH4 domain of IGF2BPs, containing an evolutionarily conserved structural element that confers m^6^A specificity [96]. IGF2BP1 has been shown to target PD-L1, accelerating immune evasion in CC by reducing CD8^+^ T cell-mediated tumour cytotoxicity in an m^6^A-dependent manner [97]. IGF2BP2 read m^6^A modifications on circQSOX1 and promoted CRC progression and immune escape through the miR-326/miR-330–5p/PGAM1 axis, which compromised anti-CTLA-4 therapy in CRC patients [98]. In pancreatic cancer, IGF2BP3 expression is positively correlated with the infiltration of immune cells such as CD4^+^ T cells, CD8^+^ T cells, macrophages, DCs, and neutrophils. Higher IGF2BP3 expression in patients with PDAC is also associated with a better response to anti-PD-1/PD-L1 therapy [99]. Furthermore, IGF2BP3 read WTAP-mediated m^6^A modifications on circCCAR1, promoting CD8^+^ T cell dysfunction and preventing the ubiquitin-mediated degradation of PD-1 in HCC [83]. Interestingly, Fusobacterium nucleatum (F.nucleatum) was found to stimulate PD-L1 expression through m^6^A modification of IFIT1, dependent on IGF2BP2/3, facilitating immune escape in CRC [100].

In brief, RBPs can recognize and bind to m^6^A-modified transcripts, which influence RNAs fate, and further investigating the involvement of RBPs in epigenetic modifications of RNAs is important for developing RBPs-based therapeutics and reversing TIME.

## 6. Therapeutic strategies for targeting RBPs to modulate the TIME

Given the involvement of RBPs in the TIME and malignant progression, targeting RBPs offers a promising therapeutic strategy for cancer treatment (Table 4).

**Table 4.** Strategies targeting RBPs to regulate the GI TIME.

	Involved RBPs	Effects on TIME	Researchers	Year
MS-444	HuR	Reduce tumor-infiltrating CD8+ cytotoxic T cells	Majumder et al.	2022 [101]
C1632	Lin28	Enhance anti-tumor cytotoxicity of GPC3-CAR T cells or NK cells	Patra T et al.,Jennings et al.	2021,2023 [39,103]
M/m-MP@F	m6A related RBPs	Increase m6A methylation and promote DC cells maturation	Xiao et al.	2023 [106]
OMV-L-mRNA (OVA-box C/D)	L7Ae	Deliver mRNA antigens into BMDCs and induce antigen presentation	Gao et al.	2023 [111]
VNP-siYTHDF1	YTHDF1	Suppress recruitment of MDSCs and improve the infiltration of CD8+ T cells to enhance anti-PD1 response and overcome ICB-based immunotherapy resistance	Bao et al.	2023 [107]
c(RGDyC)- siYTHDF1	YTHDF1	Promote expression of IFN-γ receptor 1 on tumor cells and MHCI for antigen presentation	You et al.	2023 [108]
nanoparticle-siRNA or STM2457	METT3	Reactivate CD8+ T cells and restore IFN-γ+	Chen et al.,Pan et al.	2022,2023 [109,110]
N6L	NCL	Reduce the proportion of Tregs and Gr-MDSC	Ponzo et al.	2022 [105]
SIIN-CIRP	CIRP	Induce DCs activation, enhance presentation of CIRP-bound antigens to T-cells and induce T-cells response	Villanueva L et al.	2017 [112]

Firstly, HuR is a particularly attractive target due to its significant role in modulating the TIME in GI tumours. HuR inhibitors demonstrate potential due to their cancer-selective cytotoxicity. For example, Lang et al. [101] demonstrated that the HuR inhibitor MS-444 reduced tumour-infiltrating CD8^+^ cytotoxic T cells and restored spleen architecture and Peyer patches in APC^min^ mice. Another novel compound, KH-3, also showed a strong correlation with HuR expression in PDAC, directly disrupting HuR-RNA binding and suppressing pancreatic cancer cells viability [102]. These findings highlight that targeting HuR not only improves the prognosis of patients with GI tumours but also provides a framework for targeting RBPs in cancer therapy. Additionally, small-molecule inhibitors like C1632, which target LIN28, had been shown to reduce PD-L1 expression and suppress IDO1, decreasing kynurenine production and enhancing the cytotoxicity of GPC3-CAR T cells or NK cells by inhibiting Sox2/Oct4 pathways [39,103]. It was found that RBP nucleolin (NCL) was associated with hypoxia responses and was a poor prognostic marker in PDAC [104]. One study demonstrated that N6L, a multivalent pseudopeptide, bound to NCL and inhibited its expression, reducing the proportion of Tregs and granulocytic myeloid-derived suppressor cells (Gr-MDSCs) [105]. NCL also decreased IL-6 production, thereby lowering the number of immunosuppressive cells within the TIME and promoting CD8^+^ T cells infiltration [94].

Recently, nanomedicines have emerged as an effective method for targeting RBPs. Xiao et al. [106] developed the M/m-MP@F, which encapsulated an FTO inhibitor (FB23–2) in mesoporous particulate MPDA (MP), with antigen-capturing maleimide (mal) and DC-targeting mannose on its surface. This nanodrug, after thermal ablation in HCC, co-delivered tumour-associated antigens (TAAs) and the FTO inhibitor to tumour-infiltrating dendritic cells (TIDCs), increasing m^6^A methylation and triggering a strong antitumor immune response through DC maturation, thereby inhibiting tumour growth and metastasis [106]. Additionally, nanoparticle-based delivery systems targeting oncogenic mRNAs have also shown significant therapeutic potential. Bao et al. [107] developed vesicle-like nanoparticles (VNPs) that encapsulated YTHDF1-siRNA to silence YTHDF1 expression. This system demonstrated strong antitumor effects in CRC, improving tumour response to anti-PD1 therapy and overcoming resistance to immune checkpoint blockade (ICB) by suppressing MDSC recruitment and enhancing CD8^+^ T cell infiltration [107]. Further, You et al. [108] also designed engineered small extracellular vesicles (sEVs) modified with CD47 and cyclic RGD (c(RGDyC)) to deliver siYTHDF1. This approach effectively increased IFN-γ receptor 1 expression on tumour cells and MHCI for antigen presentation, triggering a robust cytotoxic T lymphocyte response. In another study, targeting METTL3 using nanoparticle-siRNA or a METTL3-specific inhibitor (STM2457) in combination with anti-PD-1 therapy reactivated CD8^+^ T cells, restored IFN-γ^+^ production, and regressed the growth of orthotopic NAFLD-HCC tumours [109,110]. Interestingly, bacterial outer membrane vesicles (OMVs) act as natural nanomaterials for drug delivery have been explored. Gao et al. [111] engineered OMVs using a surface RNA-binding protein, L7Ae, as an mRNA delivery platform. This OMV-L-mRNA system efficiently delivered mRNA antigens into bone marrow-derived dendritic cells (BMDCs), enhancing antigen presentation.

Lastly, given that tumour-infiltrating lymphocytes are linked to improved prognosis and responsiveness to immune checkpoint inhibitors, strategies that foster tumour inflammation could enhance the efficacy of immunotherapy. Building on this concept, Villanueva et al. [112] developed a novel vaccination platform that incorporated T-cell epitope OVA (257–264) (SIINFEKL), which were recognized by CD8^+^ T cells. the vaccination platform utilized cold-inducible RNA-binding protein (CIRP), a TLR4-binding protein, but SIIN-CIRP protein could induce DCs activation, enhance antigen presentation to T cells, and stimulate a robust T cell response [112]. Considering that RBPs contribute to cancer immune evasion and are often aberrantly expressed, personalized RBP-based vaccines (including cancer or neoantigen vaccines) offer a promising approach to reactivating tumour-specific immunity. Combining these vaccines with immune checkpoint inhibitors or radiotherapy could further improve therapeutic outcomes [113].

In summary, targeting RBPs provides novel approaches to GI tumour immunotherapy by modulating the TIME. This strategy holds significant promise for enhancing the effectiveness of cancer immunotherapy. However, potential side effects, particularly due to the inhibitory effects on normal cells, must be addressed. One solution is the development of nanoparticle delivery systems to enhance tumour-specific targeting and minimize off-target effects.

## 7. Conclusions and perspectives

In recent years, the critical roles of RBPs in various cancers have gained prominence as RNA regulation in cancer biology is increasingly explored [21]. RBPs exhibit complex regulatory interactions with RNAs and play pivotal roles in maintaining RNA stability and mediating post-transcriptional modifications, which are integral to cancer development and progression. This review highlights recent findings on how RBPs regulate both mRNAs and ncRNAs, contributing to the formation of the TIME in GI tumours. However, only a subset of RBPs involved in regulating the TIME of GI tumours was discussed, and many interactions between RBPs and RNAs remain underexplored.

To advance RBPs-based targeted therapy, it is essential to identify core, cancer-specific RBPs in GI tumours that can modulate the TIME and improve immunotherapy efficacy. However, the complexity of tumour heterogeneity, inter-patient variability, and the diverse regulatory mechanisms of RBPs pose challenges to the full realization of RBPs as therapeutic targets. Conventional RNA sequences recognized by specific RBP domains still exist, underscoring the need to identify precise targets. Personalized treatment strategies, tailored to the unique RBP profiles of individual patients, will be essential for improving treatment efficacy and minimizing side effects. As biotechnology and bioinformatics continue to evolve, more RBPs will be identified and linked to TIME remodelling, providing new avenues for therapeutic intervention. Recently, various experimental approaches have been developed to map the interactions between RBPs and RNAs. One of the primary techniques is RNA Immunoprecipitation (RNA IP), which couples the immunoprecipitation of a specific RBP with quantitative RT-PCR (qPCR) to identify its associated transcripts. Additionally, high-throughput sequencing of cross-linked immunoprecipitation (HITS-CLIP) can be used to map RBP-specific binding sites across the transcriptome [114,115]. Small RNA libraries, generated using UV crosslinking and RNAse digestion, are another approach that provides comprehensive RBP binding profiles for the entire transcriptome. In contrast, RNA-centric reverse proteomics is employed to identify RBPs that bind to known cis-regulatory regions. For example, RNA ligands have been used to identify RBPs that bind to AREs in the 3’UTR of mRNAs encoding key cytokines such as TNF-alpha and IFN-γ [116].

While some RBPs-based therapies remain in pre-clinical stages, such as the HuR inhibitor MS-444, antisense oligonucleotides (ASOs) against MSI1, and siRNA-targeted drug delivery systems, these approaches show promise for treating GI tumours [117–119]. However, RBPs also play roles in drug resistance pathways and gene modifications that contribute to treatment failure. For instance, HuR mediates resistance to oxaliplatin in CRC by upregulating CDC6 expression through direct binding to its 3’UTR [120], and silencing HuR reverses epirubicin resistance in CRC [121]. YTHDF1, another RBP, promotes cisplatin resistance in CRC by targeting GLS1 and enhancing glutaminase-1 metabolism [122]. Additionally, Lin28 overexpression increases resistance to paclitaxel and 5-fluorouracil in gastric and hepatocellular carcinoma cells through its regulation of miR-107 and Let-7 [123,124]. The dysregulation of RBPs is therefore closely associated with chemoresistance in tumours. Understanding the molecular mechanisms underlying RBPs-related chemoresistance offers new opportunities for targeted therapy in GI tumours. Considering the specific expression profiles or mutational significance of RBPs in the TIME of each patient can lead to more optimized and personalized treatment strategies. Another question is that RBPs are involved in regulating transcriptional stability, but RBPs-based therapies can occur off-target effects, such as a total knockdown of HuR in cells is lethal [125]. Therefore, targeting RBPs to regulate TIME should selectively inhibit RBPs in specific cells. Although nanomedicines have great potential for clinical applications in targeting RBPs, it is necessary to further explore the bioinformation of RBPs, optimize the design parameters of the nanocarriers and introduce endogenous or exogenous stimulations, in response to the nanomaterials to achieve controlled release of the nanomaterials [126].

In conclusion, exploring the regulatory mechanisms of RBPs within the TIME of GI tumours, and optimizing RBP-based therapies, presents promising opportunities for developing novel targeted therapies and immunotherapeutic strategies for GI tumours.

## Abbreviations

ALKBH5: Alkylation repair homolog 5
AMAP1: Arf-GAP protein 1
AREs: Adenine and uridine-rich elements
ARF6: ADP-ribosylation factor 6
ASOs: Antisense oligonucleotides
BAG3: Bcl-2-associated athanogene 3
Bcl-2: B-cell lymphoma 2
BMDCs: Bone marrow-derived dendritic cells
CAFs: Cancer-associated fibroblasts
CAR: Chimeric antigen receptor ()
CC: Colon cancer
CeRNAs: Competitive endogenous RNAs
CircRNAs: Circular RNAs
CIRP: Cold-inducible RNA-binding protein
c-MYC: Cell-myc
CRC: Colorectal cancer
CTLA4: Cytotoxic T-lymphocyte-associated protein 4
DCs: Dendritic cells
eCLIP: Enhanced crosslinking and immunoprecipitation
EIF4A: Eukaryotic translation initiation factor 4a
EIF4E: Eukaryotic translation initiation factor 4e
EZH2: Zeist homologous enhancer 2
F.nucleatum: Fusobacterium nucleatum
GC: Gastric cancer
GI: Gastrointestinal
Gr-MDSCs: Granulocytic myeloid-derived suppressor cells
HCC: Hepatocellular carcinoma
HITS-CLIP: High-throughput sequencing of cross-linked immunoprecipitation
HITT: HIF-1α inhibitor
HMGA2: High mobility group A2
HMGB1: High mobility group box 1
HO-1: Heme oxygenase-1
HSF1: Heat Shock Transcription Factor 1
HuR: Human antigen R
IAP1: Apoptosis protein 1
IAP2: Inhibitor of apoptosis protein 2
ICAM-1: intercellular adhesion molecule-1
ICB: Checkpoint blockade
ICC: Intrahepatic cholangiocarcinoma
IDO1: Indoleamine 2,3-dioxygenase-1
IFNγ: interferon-gamma
IL-10: Interleukin 10
IL-1β: Interleukin 1 beta
IMP3: Insulin like growth factor 2 mRNA-binding protein 3
IRAKM: Interleukin-1 Receptor-Associated Kinase 3
IRF1: Interferon regulatory factor 1
KH: K homology
KRAS: Kirsten rat sarcoma virus
LIN28A/B: Lin-28 homologs A/B
LncRNAs: Long non-coding RNAs
LPS: Lipopolysaccharide
m6A: N6-methyladenosine
MDSCs: Myeloid-derived suppressor cells
METTL3: Methyltransferase 3
MICB: MHC class I polypeptide related sequence B
MIR155HG: MIR155 host gene
MiRISC: MiRNA-induced silencing complex
MiRNAs: MicroRNAs
MREs: MiRNA response elements
MSI: Musashi
MSI2: Musashi-2
NCL: Nucleolin
ncRNAs: Noncoding RNAs
NK: Natural killer
NKG2D: Natural killer group 2 member D
NPM3: Nucleoplasmin3
OCT4: Octamer-binding transcription factor 4
OMVs: Outer membrane vesicles
PBMLs: Peripheral blood mononuclear lymphocytes
PDAC: Pancreatic ductal adenocarcinoma
PIK3R1: Phosphoinositide 3 kinase regulatory subunit 1
PKM2: RBP pyruvate kinase M2
PTRH1: Peptidyl-tRNA hydrolase 1 homolog
PUM: Pumilio
q-PCR: Quantitative RT-PCR
RBDs: RNA-binding domains
RBPs: RNA-binding proteins
RTK: Receptor tyrosine kinase
SOX2: Sex-determining region Y-box 2 ()
STAT1: Signal transducer and activator of transcription 1
STING: Stimulator of interferon genes
TAAs: Tumour-associated antigens
TAMs: Tumour-associated macrophages
TDEs: Tumour-derived exosomes
TGFβ: Transforming growth factor beta
TIDCs: Tumour-infiltrating dendritic cells
TIME: Tumour immune microenvironment
TLR: Toll like receptor
TME: Tumour microenvironment
TNFR: TNF receptor
TNFα: Tumour necrosis factor-alpha
TNTs: Tunneling nanotubes
Treg: Regulatory T
TTP: Tristetraprolin
ULBP2: UL16-binding protein 2
VNPs: Vesicle-like nanoparticles
WTAP: Wilms tumour 1-associating protein
YB1: YBX1
YTHDF1: YTH N6-methyladenosine RNA binding protein 1

## Data Availability

No datasets were generated or analysed during the current study.
